# Differentiation of hepatocellular adenoma by subtype and hepatocellular carcinoma in non-cirrhotic liver by fractal analysis of perfusion MRI

**DOI:** 10.1186/s13244-022-01223-6

**Published:** 2022-04-28

**Authors:** Florian Michallek, Riccardo Sartoris, Aurélie Beaufrère, Marco Dioguardi Burgio, François Cauchy, Roberto Cannella, Valérie Paradis, Maxime Ronot, Marc Dewey, Valérie Vilgrain

**Affiliations:** 1 Department of Radiology, Charité – Universitätsmedizin Berlin, Corporate Member of Freie Universität Berlin, Humboldt-Universität Zu Berlin, and Berlin Institute of Health, Charitéplatz 1, 10117 Berlin, Germany; 2 Université de Paris, CRI, U1149 Paris, France; 3 Department of Radiology, Hôpital Beaujon, AP-HP.Nord, 100 Boulevard du Général Leclerc, 92110 Clichy, France; 4 Department of Pathology, Hôpital Beaujon, AP-HP.Nord, 100 Boulevard du Général Leclerc, 92110 Clichy, France; 5 Department of HBP Surgery, Hôpital Beaujon, AP-HP.Nord, 100 Boulevard du Général Leclerc, 92110 Clichy, France; 6 Section of Radiology - BiND, University Hospital “Paolo Giaccone”, Via del Vespro 129, 90127 Palermo, Italy; 7 Department of Health Promotion Sciences Maternal and Infant Care, Internal Medicine and Medical Specialties, PROMISE, University of Palermo, 90127 Palermo, Italy; 8 DKTK (German Cancer Consortium), Partner Site, Berlin, Germany

**Keywords:** Hepatocellular adenoma, Hepatocellular carcinoma, Magnetic resonance imaging, Perfusion, Fractals

## Abstract

**Background:**

To investigate whether fractal analysis of perfusion differentiates hepatocellular adenoma (HCA) subtypes and hepatocellular carcinoma (HCC) in non-cirrhotic liver by quantifying perfusion chaos using four-dimensional dynamic contrast-enhanced magnetic resonance imaging (4D-DCE-MRI).

**Results:**

A retrospective population of 63 patients (47 female) with histopathologically characterized HCA and HCC in non-cirrhotic livers was investigated. Our population consisted of 13 hepatocyte nuclear factor (HNF)-1α-inactivated (H-HCAs), 7 β-catenin-exon-3-mutated (b^ex3^-HCAs), 27 inflammatory HCAs (I-HCAs), and 16 HCCs. Four-dimensional fractal analysis was applied to arterial, portal venous, and delayed phases of 4D-DCE-MRI and was performed in lesions as well as remote liver tissue. Diagnostic accuracy of fractal analysis was compared to qualitative MRI features alone and their combination using multi-class diagnostic accuracy testing including kappa-statistics and area under the receiver operating characteristic curve (AUC). Fractal analysis allowed quantification of perfusion chaos, which was significantly different between lesion subtypes (multi-class AUC = 0.90, *p* < 0.001), except between I-HCA and HCC. Qualitative MRI features alone did not allow reliable differentiation between HCA subtypes and HCC (κ = 0.35). However, combining qualitative MRI features and fractal analysis reliably predicted the histopathological diagnosis (κ = 0.89) and improved differentiation of high-risk lesions (i.e., HCCs, b^ex3^-HCAs) and low-risk lesions (H-HCAs, I-HCAs) from sensitivity and specificity of 43% (95% confidence interval [CI] 23–66%) and 47% (CI 32–64%) for qualitative MRI features to 96% (CI 78–100%) and 68% (CI 51–81%), respectively, when adding fractal analysis.

**Conclusions:**

Combining qualitative MRI features with fractal analysis allows identification of HCA subtypes and HCCs in patients with non-cirrhotic livers and improves differentiation of lesions with high and low risk for malignant transformation.

**Supplementary Information:**

The online version contains supplementary material available at 10.1186/s13244-022-01223-6.

## Key points

- Fractal analysis quantifies chaos of perfusion in different hepatocellular adenoma (HCA) subtypes and hepatocellular carcinoma (HCC) in the non-cirrhotic liver using perfusion MRI.
- Visual analysis of MRI features was not sufficiently reliable to differentiate between HCA subtypes and HCCs in non-cirrhotic livers.
- Combining fractal analysis of perfusion and qualitative MRI features allowed reliable prediction of the histopathological lesion subtype.
- Fractal analysis of perfusion improved differentiation of lesions by risk for malignant transformation.

## Background

Hepatocellular adenomas (HCAs) are rare liver tumors that mainly develop in young women taking oral contraception [1]. HCAs are a heterogeneous group of different subtypes of neoplastic benign hepatocellular proliferations. Genotype–phenotype classifications have led to the identification of five distinct subtypes based on morphological and immunophenotypical features, which are currently used in clinical practice: hepatocyte nuclear factor (HNF)-1α-inactivated HCA (H-HCA), inflammatory HCA (I-HCA), β-catenin-exon-7/8-mutated HCA (b^ex7/8^-HCA), β-catenin-exon-3-mutated HCA (b^ex3^-HCA), sonic hedgehog HCA (shHCA), and unclassified HCA, with two mixed forms derived from I-HCA and the two variants of b-HCA [2–4]. The most frequent subtypes are H-HCA (30%-40% of all HCAs) and I-HCA (35–45% of all HCAs) [1]. Histologically, H-HCAs are characterized by the presence of steatosis, I-HCA feature pseudo-portal tracts with inflammation, large arteries, ductular reaction, and sinusoidal dilatation and congestion, b-HCAs show cytological atypias, small-cell liver changes, a pseudoglandular/acinar architecture, and cholestasis, and shHCAs present with hemorrhage [5].

The European Association for the Study of the Liver (EASL) issued recommendations for the management of HCA [6], acknowledging that the risk of complications, such as malignant transformation and bleeding, is strictly influenced by sex and tumor size [7]. Aside from that, subtyping of HCAs should also be considered since different subtypes are associated with different outcomes [3]. Indeed, the risk of malignant transformation into hepatocellular carcinoma (HCC) is higher for the b-HCA subtype, reaching 40% in these HCAs, whereas H-HCAs have a low potential for malignant transformation [8]. I-HCAs have been proven to have a higher probability of regression during follow-up [9].

Magnetic resonance imaging (MRI) has shown potential for HCA subtyping, using combinations of features associated with different tumor phenotypes [10, 11]. Sensitivities and specificities around 90% have been reported for differentiating the two most common subtypes, H-HCA and I-HCA [12]. However, some features depend on the employed contrast agent, e.g., the presence of an enhancing capsule, which is not entirely characteristic to HCC, since a peripheral pseudocapsule has also been noticed in HCA [13]. While results for the differentiation of β-catenin-mutated HCA and HCC in non-cirrhotic liver have not been consistent using imaging alone, high specificity has been reported for the hepatobiliary contrast agent phase [14]. Therefore, biopsy is a common requirement in the clinical setting [15, 16]. To date, qualitative interpretation of MRI features, enhancement patterns, and nodule appearance on hepatobiliary phase images are the cornerstones of imaging-based characterization of HCAs [10]. Few studies have investigated quantitative interpretation of imaging data [17]. Notably, no attempts have been reported to quantitatively assess perfusion patterns of HCA and HCC.

Perfusion is inherently chaotic, and perfusion patterns tend to vary with the underlying biological tissue characteristics and vascular structure. Fractal analysis has been established as a method to quantitatively assess perfusion chaos by calculating the fractal dimension (FD) [18], which can be interpreted as a quantitative measure of chaos [19, 20]. Vascular structure can be interpreted as an anatomical hallmark that determines the observable perfusion pattern. Thus, quantitative assessment of the perfusion pattern might allow conclusions to be drawn on the underlying vascular structure.

The objective of this proof-of-concept study was to investigate the feasibility and diagnostic performance of fractal analysis of perfusion using 4D dynamic contrast-enhanced (DCE) MRI to differentiate between subtypes of HCA and HCC in the challenging subgroup of non-cirrhotic patients.

## Methods

### Patients

**Fig. 1:**
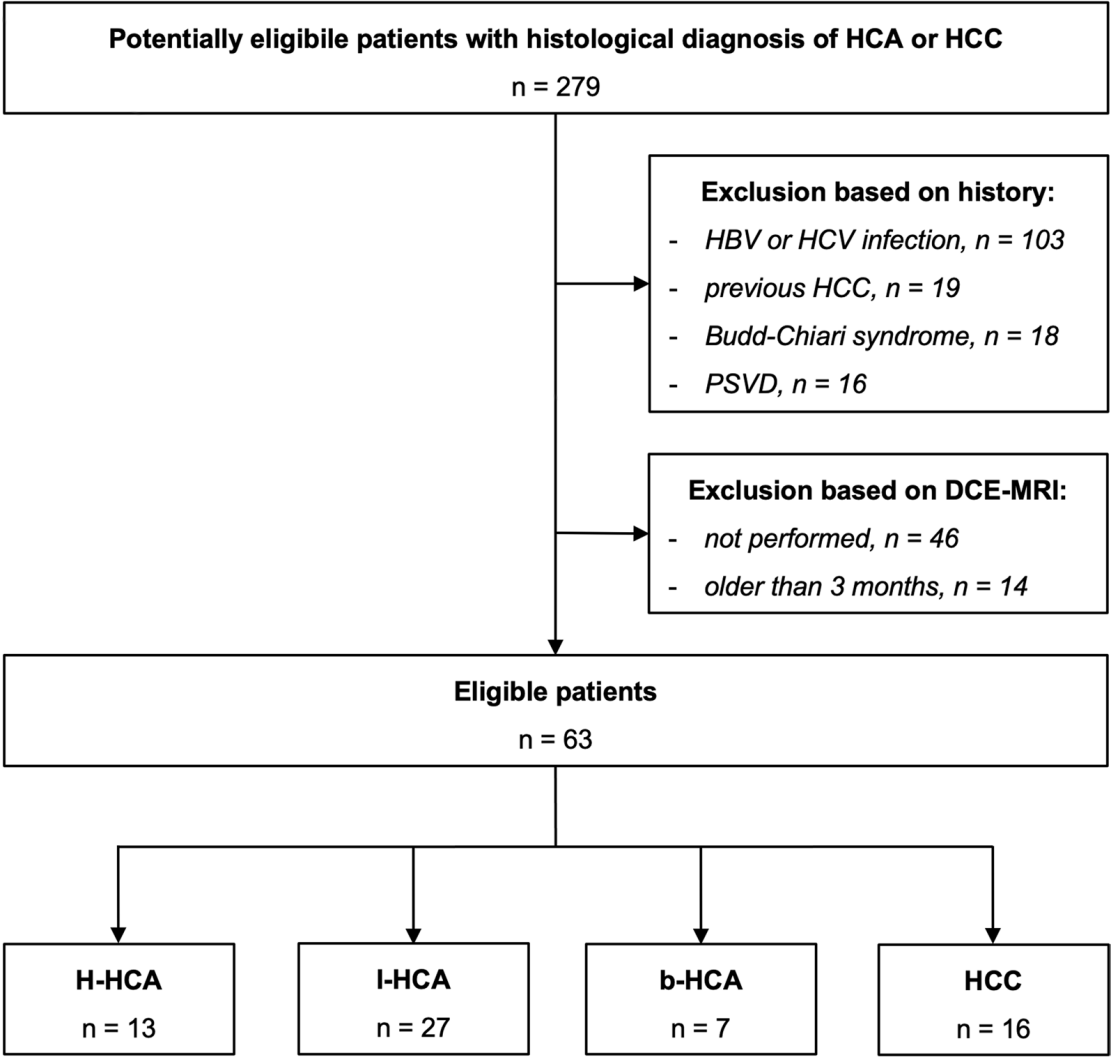
Flowchart of retrospective patient inclusion. HCA—hepatocellular adenoma, HCC—hepatocellular carcinoma, HBV/HCV—hepatitis B or C virus, PSVD—porto-sinusoidal venous disease, DCE-MRI—dynamic contrast-enhanced magnetic resonance imaging, H-HCA—HNF1α-inactivated HCA, b-HCA—β-catenin-exon-3-mutated HCA, I-HCA—inflammatory HCA

Consecutive patients with histologically characterized and subtyped HCA or HCC and non-cirrhotic hepatic parenchyma (i.e., fibrosis class F0-F1 in histological analysis) examined at Beaujon Hospital in Paris, France, between January 2015 and December 2020 were retrospectively analyzed. Inclusion criteria were patients who underwent liver MRI within 3 months of histological confirmation and lesion diameter of 2 cm or greater. Exclusion criteria were subjacent liver disease (i.e., Budd–Chiari syndrome, non-cirrhotic portal hypertension, hereditary hemochromatosis, non-alcoholic fatty liver disease), presence of hepatitis B or C virus infection, previous HCC, previous systemic or locoregional treatments of the lesion, or hemorrhagic presentation of the lesion. When multiple nodules were present, one histologically confirmed lesion per patient was analyzed, which corresponded to either the largest, best accessible, or most conspicuous lesion, respectively. Histological diagnosis of both lesion and liver parenchyma was obtained by percutaneous biopsy or by analysis of resected liver specimens. For each lesion, we compiled the results of immunohistochemical analysis and, where performed, of molecular analysis, according to the recent genotype–phenotype classification (Additional file 1: Table S1) [3, 21]. In case of diagnostic doubt, molecular analysis was performed. Clinical data, results of laboratory tests, and hemodynamic data were collected. Institutional review board approval was obtained for this observational retrospective study, and informed written consent for patient inclusion was waived. Figure 1 shows the flowchart of patient selection.

### Imaging

Patients were examined using the standard clinical liver MRI protocol on one of two clinical MRI scanners (1.5 T SIGNA Artist, GE Healthcare; 3 T Achieva, Philips Healthcare) equipped with high-performance gradients and phased-array coils. After acquisition of pre-contrast sequences, 0.05 mmol/kg body weight of Gd-BOPTA (MultiHance, Bracco Imaging) or Gd-DOTA (Dotarem, Guerbet) followed by a 20 mL saline solution flush was administered at 2 and 1 mL/s, respectively, with a power injector. A multi-arterial phase, a portal venous phase, and a three-minute delayed phase were acquired. Technical details of the MRI protocol are provided in Additional file 1: Table S2.

### Qualitative analysis of MRI features

Qualitative analysis of MRI features was performed by two radiologists (R.S. and M.D.B., 6 and 10 years of experience in abdominal radiology) in consensus according to typical imaging appearance by EASL guidelines [6] and evaluated, e.g., in [10, 22, 23]. In summary, typical H-HCA imaging characteristics include non-rim arterial phase hyperenhancement (APHE), non-rim washout, and homogeneous signal drop on opposed-phase compared to in-phase T1-weighted gradient echo images. I-HCA typically shows non-rim APHE, hyperenhancement during the portal venous and delayed phase, and marked T2 hyperintensity, with the “atoll sign” being highly specific for I-HCA. Typical features for HCC include non-rim APHE, non-rim washout in the portal venous or delayed phases and presence of an enhancing capsule. However, for b^ex3^-HCAs no typical imaging features have been consistently agreed upon in the literature and are not reliably differentiable from HCCs [6]. Therefore, any lesions without typical features were considered “undetermined from imaging” and statistically treated as non-diagnostic, as explained below.

### Image preprocessing

Image preprocessing was performed prior to fractal analysis and consisted of the following steps: multi-phase registration, image denoising, intensity standardization, and segmentation. All image preprocessing is detailed in Additional file 1: Image Preprocessing.

### Fractal analysis

Branching patterns of the vascular tree are a multi-scale phenomenon and feature fractal structure [24]. Perfusion, the physiological process corresponding to vascular anatomy, mirrors the anatomical fractal properties such that the fractal phenotype of the perfusion pattern depends on the underlying anatomy of the vascular tree [25]. Perfusion patterns have previously been characterized by fractal analysis of radiological and nuclear medicine imaging datasets with fractal dimension (FD) as quantitative imaging biomarker [19]. FD has been shown to quantify chaos of perfusion patterns, thereby conveying pathophysiologically relevant information on vascular structure and function [20]. In this study, we applied fractal analysis to dynamic contrast-enhanced MRI datasets. We calculated FD maps to visualize the local amount of perfusion chaos, and we hypothesized that characterizing perfusion patterns by fractal analysis would allow conclusions to be drawn on differences in underlying vascular anatomy between hepatocellular adenomas and carcinomas. To this end, a previously established fractal analysis method [26, 27] was applied to 4D DCE-MRI to generate (1) a local map of the FD and to derive (2) a global FD value for the whole liver lesion as well as for representative sites in remote normal liver tissue similar to the ones used in [28]. We applied fractal analysis in a full 4D manner comprising an arterial, portal venous, and delayed phase of contrast agent distribution. We created maps of the local FD for visual inspection, and we calculated the global FD for each liver lesion and a representative site in adjacent liver parenchyma. We subtracted the global FD of normal liver parenchyma from the global FD of the lesion to obtain the FD difference (FD_diff_), thereby accounting for individual levels of perfusion chaos and standardizing the measurement.

### Statistical analysis

We used the two-sided Student’s t test to identify clinical differences in the patient population or classical MRI features. Descriptive characteristics of liver lesions are expressed as mean ± standard deviation or median with quartiles or range, where applicable. The local FD maps were analyzed qualitatively as well as quantitatively using histogram statistics including calculation of histogram skewness and kurtosis. Global FD differences (FD_diff_) were assessed using descriptive statistics. The Kruskal–Wallis test and pairwise comparisons with the Wilcoxon test were used to identify FD differences for each lesion type (i.e., each HCA subtype and HCC). Optimal cutoff values were determined by Youden’s J-index. Overall diagnostic accuracy was assessed in terms of the multi-class analysis of the area under the receiver operating characteristic curve (AUC) with confidence intervals being computed by bootstrapping as in [29]. Quadratic-weighted κ-statistics were calculated to compare agreement of visual analysis and fractal analysis with the histopathological reference standard. For differentiating lesion types by clinical relevance, we divided the lesions in our study population into a high-risk group (HCCs and b^ex3^-HCAs) and a low-risk group (H-HCAs and pure I-HCAs) according to malignancy or, respectively, potential to undergo malignant transformation. Non-diagnostic lesions were handled in an intention-to-diagnose approach as explained in [30], by assigning non-diagnostic high-risk lesions to the diagnosed low-risk group and non-diagnostic low-risk lesions to the diagnosed high-risk group. This approach ensures a more realistic evaluation in non-diagnostic cases [30]. Inter-reader agreement was assessed by unweighted κ-statistics and Bland–Altman analysis. A level of *p* ≤ 0.05 after Bonferroni correction, where applicable, was considered statistically significant. The STARD criteria for studies reporting on diagnostic accuracy were adhered to. Statistical analysis was performed using R (v3.4.1).

## Results

### Patient population

From a total of 279 patients screened, 63 patients were eligible according to our inclusion criteria (47 female [75%], mean age 41 ± 12 years, range 18–79, see Fig. 1; Tables 1, 2). Our study population included 16 HCC patients and 47 HCA patients. The HCA group consisted of 13 H-HCAs, 7 b^ex3^-HCAs, and 27 I-HCAs. Mean lesion size was larger in HCC than in HCA (81 ± 26 mm vs. 58 ± 25 mm, *p* = 0.003). The final histopathological diagnosis was based on surgical specimens in 27 patients (HCC: 13, HCA: 14) and biopsy in the remaining cases.

**Table 1:** Patient characteristics

	All *n* = 63	HCC *n* = 16	HCA *n* = 47	*P* value
Women (%)	47 (75)	4 (25)	43 (92)	** < .001**
Age (years) ± SD (range)	41 ± 12 (18–79)	58 ± 14 (29–79)	35 ± 7 (18–57)	** < .001**
BMI (kg/m^2^) ± SD	25 ± 6	25 ± 5	26 ± 3	.34
Days between MRI and histology ± SD (range)	41 ± 25 (0–95)	34 ± 25 (1–85)	43 ± 25 (0–95)	.22
Lesion size (mm) ± SD (range)	64 ± 28 (20–163)	81 ± 26 (38–163)	58 ± 25 (20–143)	**.003**
Surgical resection (%)	27 (43)	13 (81)	14 (30)	** < .001**
Presence of hepatic steatosis (%)^a^	21 (33)	5 (31)	16 (34)	**.02**
Presence of intralesional fat (%)^a^	20 (32)	3 (19)	17 (36)	.18

HCC, hepatocellular carcinoma; HCA, hepatocellular adenoma; BMI, Body Mass Index; SD, standard deviation; bold typeface indicates statistical significance (*p* ≤ 0.05)

^a^Pathology finding

**Table 2:** Characteristics of hepatocellular adenoma (HCA) subtypes

	All HCA (*n* = 47)	H-HCA (*n* = 13)	b-HCA (*n* = 7)	I-HCA (*n* = 27)	*P* value
Women (%)	43 (92)	12 (92)	5 (71)	26 (96)	.11
Age (years) ± SD (range)	35 ± 7 (18–57)	37 ± 8 (18–57)	32 ± 8 (22–51)	35 ± 6 (24–47)	.38
BMI (kg/m^2^) ± SD	26 ± 3	24 ± 2	25 ± 2	27 ± 3	.62
Oral estrogen intake^a^ (%)	23 (53)	5 (42)	3 (60)	15 (58)	.56
Previous pregnancy^a^ (%)	13 (30)	7 (58)	0 (0)	6 (23)	**.03**
Menopause^a^ (%)	1 (2)	1 (8)	0 (0)	0 (0)	.27
Metabolic syndrome (%)	5 (11)	1 (8)	0 (0)	4 (15)	.56
Days between MRI and histology ± SD (range)	43 ± 25 (0–90)	43 ± 30 (0–90)	60 ± 21 (2–90)	39 ± 25 (0–89)	.28
Lesion size (mm) ± SD (range)	58 ± 25 (20–143)	60 ± 20 (25–143)	73 ± 29 (38–125)	54 ± 25 (20–140)	.21
Surgical resection (%)	14 (30)	2 (15)	5 (72)	7 (26)	**.03**
Presence of hepatic steatosis (%)^b^	16 (34)	1 (8)	1 (14)	14 (52)	**.01**
Presence of intralesional fat (%)^b^	17 (36)	12 (92)	4 (57)	1 (4)	** < .001**
MRI features (%)					
Arterial phase					
Hyperintense	38 (81)	8 (62)	5 (71)	25 (93)	**.05**
Isointense	7 (15)	4 (31)	1 (14)	2 (7)	.15
Hypointense	2 (4)	1 (8)	1 (14)	0 (0)	.42
Portal venous phase					
Hyperintense	24 (51)	0 (0)	1 (14)	23 (85)	** < .001**
Isointense	8 (17)	2 (15)	3 (43)	3 (11)	.14
Hypointense	15 (32)	11 (85)	3 (43)	1 (4)	** < .001**
Delayed phase					
Hyperintense	22 (47)	0 (0)	1 (14)	21 (78)	** < .001**
Isointense	6 (13)	0 (0)	2 (29)	4 (15)	.33
Hypointense	19 (40)	13 (100)	4 (57)	2 (7)	** < .001**
T2-weighted					
Hyperintense	23 (49)	1 (8)	5 (71)	17 (63)	**.001**
Isointense	16 (34)	7 (54)	2 (29)	7 (26)	.21
Hypointense	8 (17)	5 (39)	0 (0)	3 (11)	.10
Out-of-phase sequence signal drop	15 (32)	10 (77)	3 (43)	2 (7)	** < .001**

HCA, hepatocellular adenoma; H-HCA, HNF1α-inactivated HCA; b^ex3^-HCA, β-catenin-exon-3-mutated HCA; I-HCA, inflammatory HCA; HCC, hepatocellular carcinoma; BMI, Body Mass Index; SD, standard deviation; bold typeface indicates statistical significance (*p* ≤ 0.05)

^a^Percentage of female population only

^b^Pathology finding

### Histopathological characteristics

Histopathological analysis identified similar prevalence of hepatic steatosis in patients with HCA and HCC (*p* = 0.20). However, in the HCA subgroup, patients with I-HCA had a higher prevalence of hepatic steatosis than patients with b^ex3^-HCA or H-HCA (*p* = 0.001). Presence of intralesional fat was not significantly different between HCC and HCA (*p* = 0.18); however, in the HCA subgroup, intralesional fat was significantly more common in patients with H-HCA than in patients with I-HCA or b^ex3^-HCA (*p* = 0.001). Histopathological findings are summarized in Tables 1 and 2, and full immunohistochemical and molecular characteristics on the per-lesion level are provided for HCA in Additional file 1: Table S1.

### Qualitative MRI features

Qualitative MRI features were significantly different among HCA subtypes for contrast enhancement characteristics (non-rim APHE, *p* = 0.05; non-rim washout, *p* < 0.001), and signal drop on the opposed-phase gradient echo sequence (*p* < 0.001). H-HCA showed a typical MRI pattern in 6/13 cases (46%). In the I-HCA subgroup, 13/27 lesions (48%) showed a typical MRI pattern, and an “atoll sign” was found in 10 of these 13 lesions. In the HCC group, 14/16 lesions (87%) with a typical MRI pattern were found. The two non-correctly categorized HCC lesions did not show APHE and hence, did not meet the predefined typical HCC criteria, although washout and a peripheral enhancing capsule on delayed phase was present. MRI features in b^ex3^-HCA were similar to those found in HCC (non-rim APHE and non-rim washout) in 4/7 patients (67%). The details are given in Table 2.

### Local fractal analysis

**Fig. 2:**
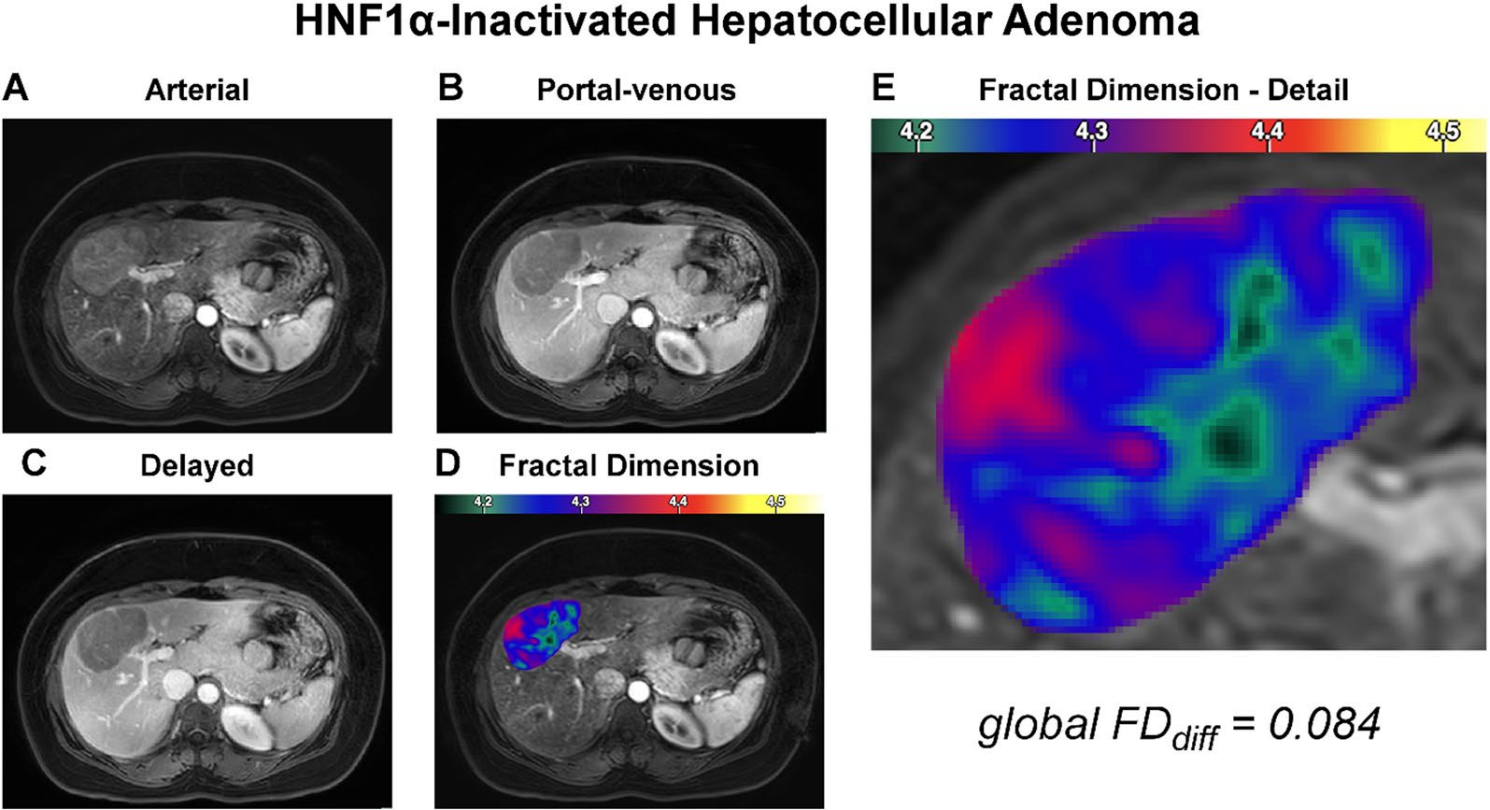
Representative case of hepatocyte nuclear factor (HNF)-1α-inactivated hepatocellular adenoma. Dynamic contrast-enhanced MR images of the arterial (**A**), portal venous (**B**), and delayed (**C**) phase are shown along with the fractal dimension (FD) map as overlay over the arterial phase (**D**) and a zoom on the lesion (**E**). The panel organization is the same throughout Figs. 2, 3, 4, and 5. The depicted patient was a 26-year-old female with obesity (BMI = 28 kg/m^2^) and no history of oral contraception. She was admitted for characterization of an incidentally found liver mass. Typical MRI features including arterial phase hyperenhancement, washout, and out-of-phase signal drop (not shown) suggested HNF1α-inactivated adenoma, which was subsequently confirmed by biopsy

**Fig. 3:**
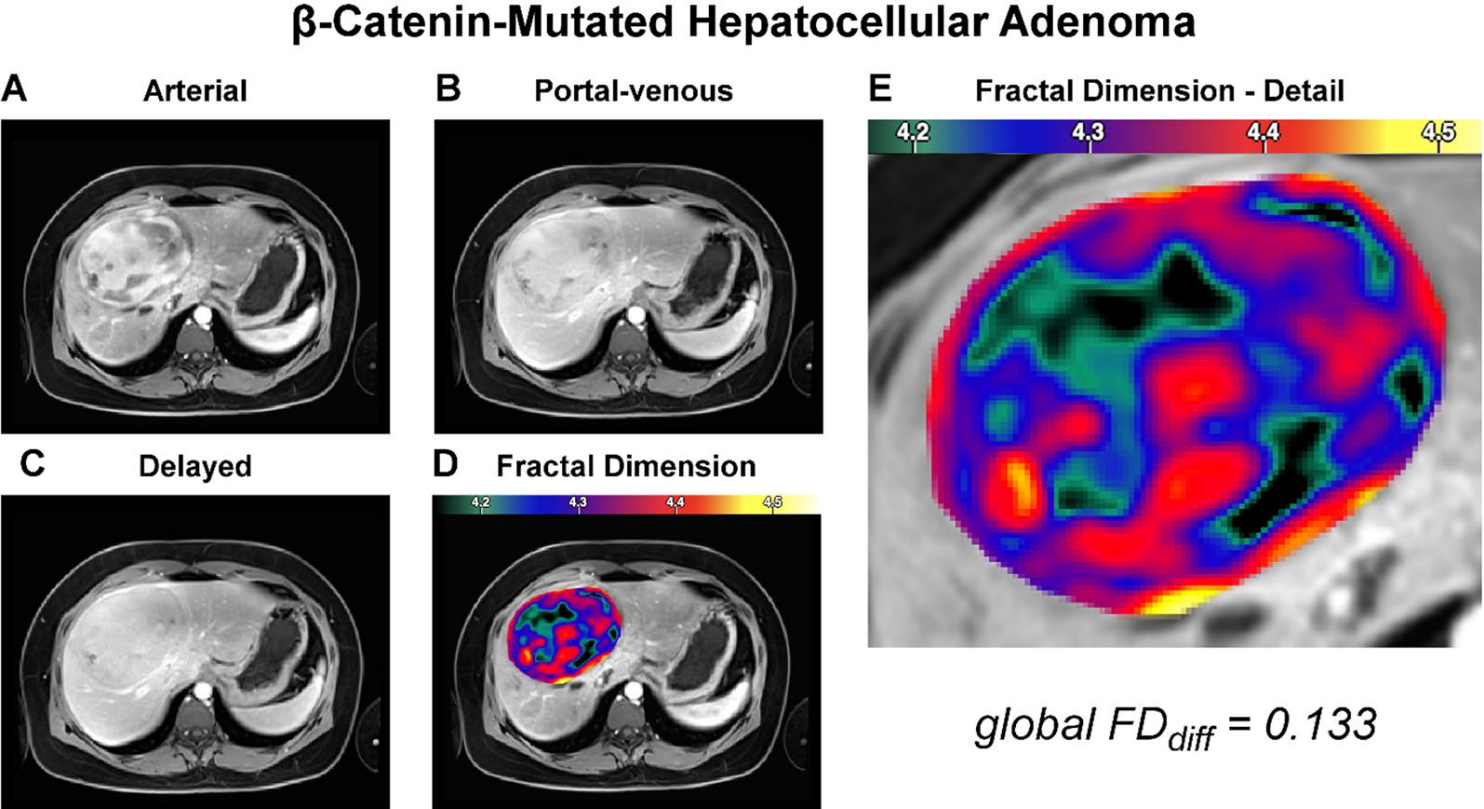
Representative case of β-catenin-exon-3-mutated hepatocellular adenoma. The panel organization is the same throughout Figs. 2, 3, 4, and 5. The depicted patient was a 34-year-old female with a 9-cm painful mass in the right liver lobe, showing arterial phase hyperenhancement without clear washout. Subsequent biopsy established the diagnosis of β-catenin-exon-3-mutated adenoma. The lesion was surgically resected

**Fig. 4:**
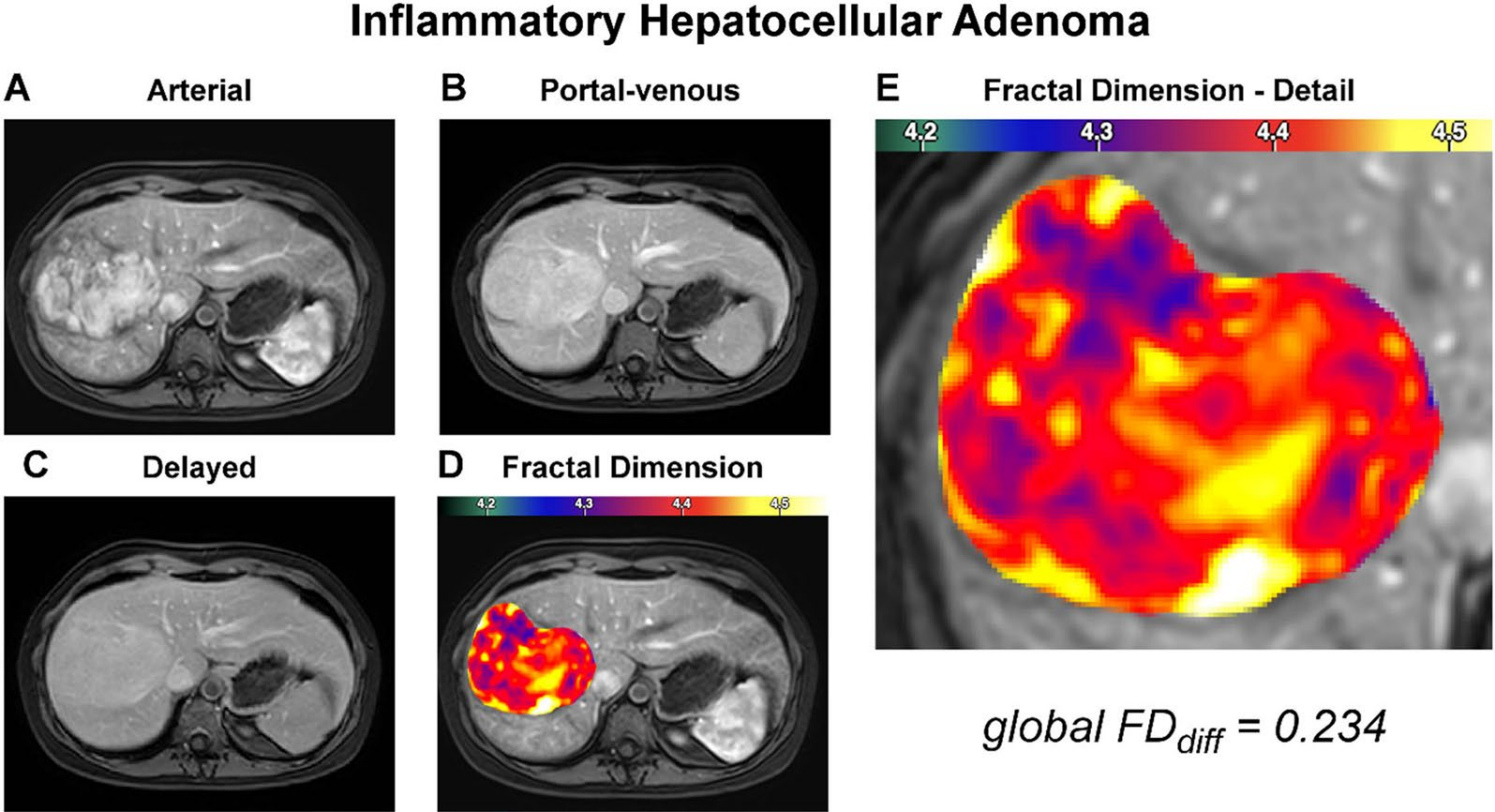
Representative case of inflammatory hepatocellular adenoma. The panel organization is the same throughout Figs. 2, 3, 4, and 5. The depicted patient was a 25-year-old female with a liver mass incidentally discovered during exploration for a chronic inflammatory state. Arterial phase hyperenhancement, persistent hyperintensity during the portal and delayed phases, and marked T2 hyperintensity (not shown) suggested inflammatory adenoma, which was confirmed by biopsy

**Fig. 5:**
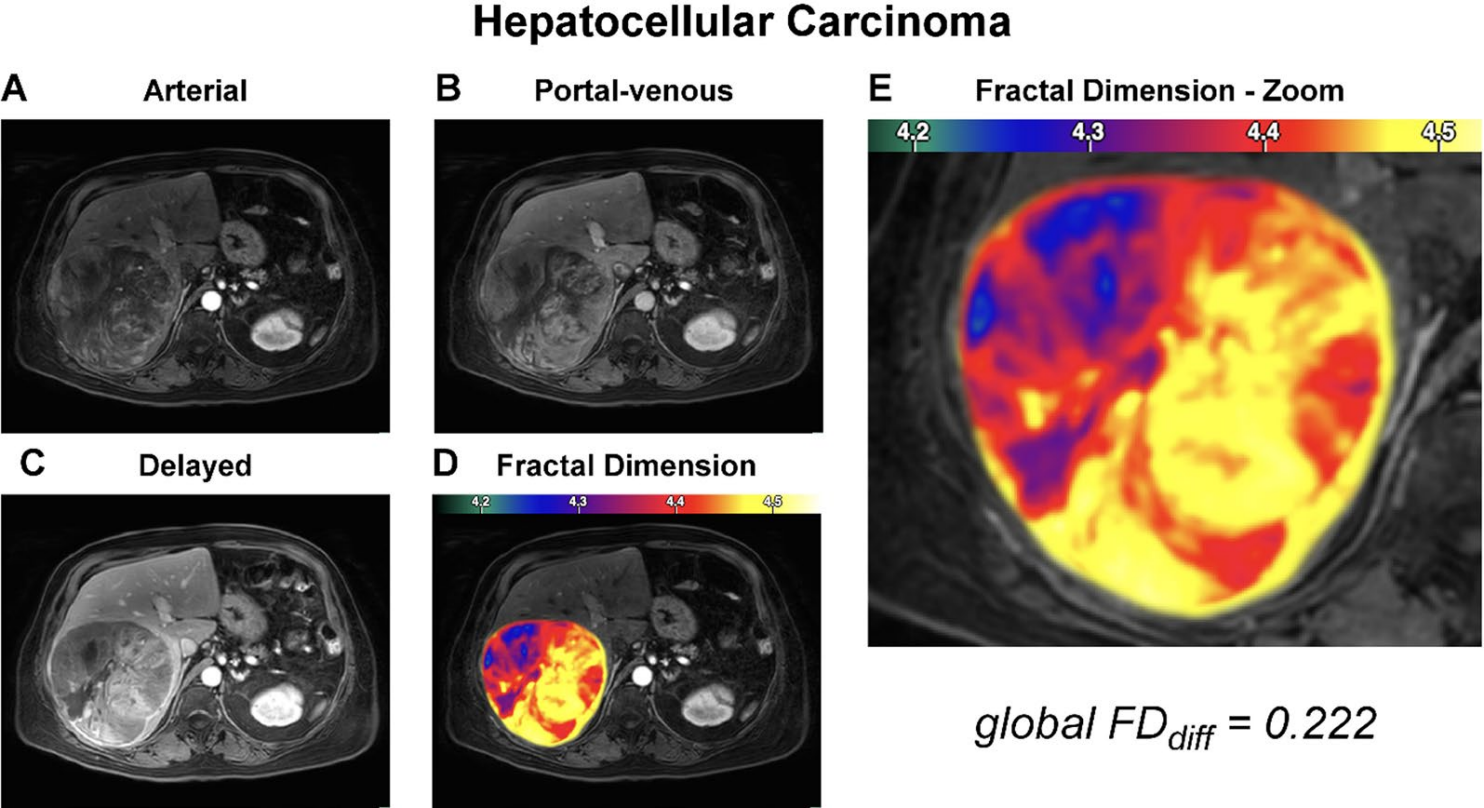
Representative case of hepatocellular carcinoma. The panel organization is the same throughout Figs. 2, 3, 4, and 5. The depicted patient was a 42-year-old man without symptoms, admitted for exploration of a liver lesion and elevated alpha-fetoprotein. MRI features with arterial phase hyperenhancement, washout, and enhancing capsule suggested hepatocellular carcinoma, confirmed by biopsy and subsequently resected specimen. Remote liver tissue showed no fibrosis

Visual inspection of MR images revealed different levels of chaos of the investigated tumor entities (Figs. 2, 3, 4, 5). In quantitative measurements, local FD was particularly high in areas with highly dynamic contrast enhancement characteristics, both spatially and temporally (see HCC example in Fig. 5). Histogram analysis of FD distributions showed that those highly chaotic regions were especially prevalent in HCCs, which had a significantly lower skewness than HCAs (skew_HCC_ = − 0.14 vs. skew_HCA_ = 0.06, *p* = 0.02). Kurtosis nonsignificantly tended to be higher in HCCs (kurt_HCC_ = 0.06) than HCAs (kurt_HCA_ = − 0.2), *p* = 0.18.

Inter-reader agreement was high (κ = 0.95, 95% confidence interval [CI] 0.90–1.0), and Bland–Altman plotting showed no relevant bias and acceptable limits of agreement (− 0.07 to 0.07).

### Global fractal analysis

**Fig. 6:**
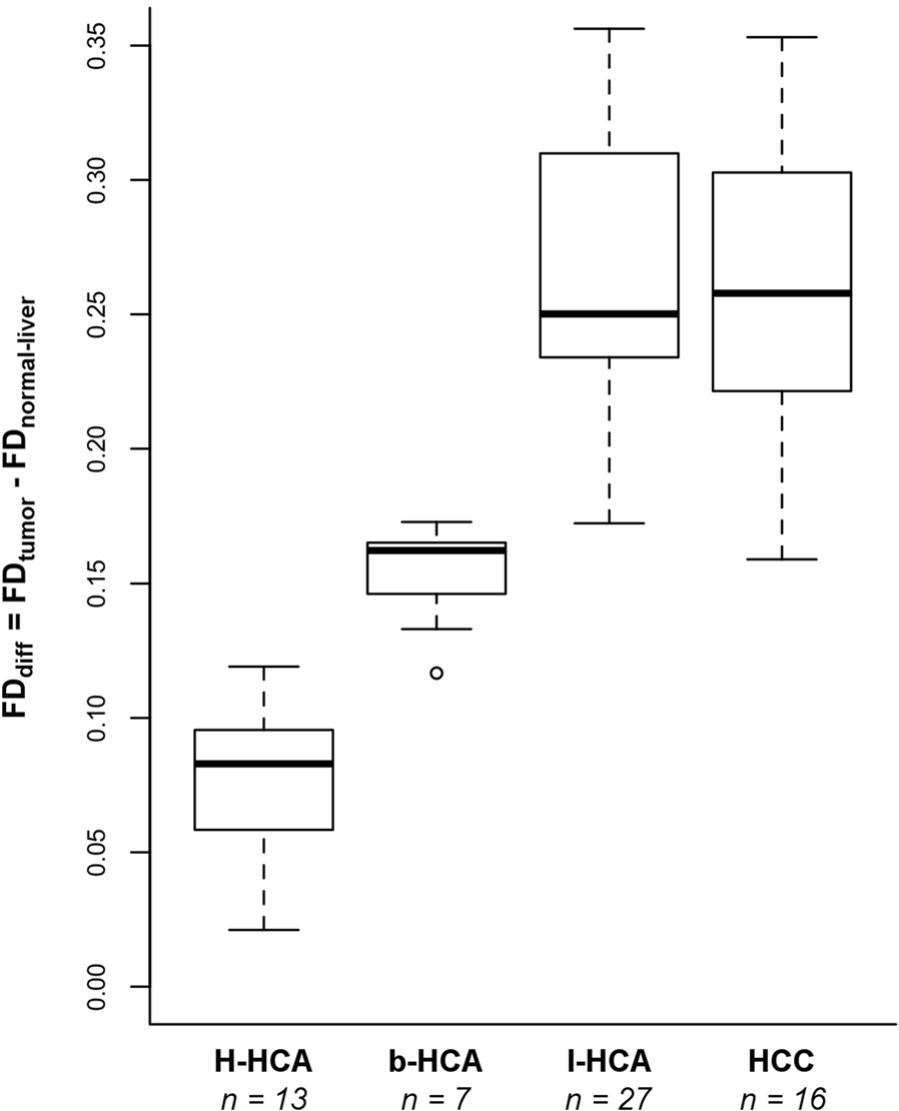
Global fractal dimension differences (FD_diff_ = FD_tumor_ − FD_normal-liver_) by tumor entity. All group comparisons were significant (*p* < 0.001) except for inflammatory adenomas (I-HCAs) versus hepatocellular carcinomas (HCCs), *p* = 1. H-HCA, HNF1α-inactivated adenomas; b^ex3^-HCA, β-catenin-exon-3-mutated adenomas

Global FD_diff_ values (FD_diff_ = FD_tumor_-FD_liver_) for HCA subtypes and HCCs were as follows: H-HCA: FD_diff_ = 0.08 (CI 0.06–0.10), b^ex3^-HCA: FD_diff_ = 0.16 (CI 0.15–0.17), I-HCA: FD_diff_ = 0.25 (CI 0.23–0.31), and HCC: FD_diff_ = 0.26 (CI 0.22–0.30); see Fig. 6 and Table 3. Global FD_diff_ values were significantly different between individual HCA subtypes and HCCs (*p* < 0.001), except between HCCs and I-HCAs, both showing a similar FD_diff_ distribution. Optimal FD_diff_ cutoff values were 0.11 and 0.18 for differentiating H-HCA, b^ex3^-HCA, and I-HCA/HCC, respectively. These cutoffs achieved a multi-class AUC of 0.90 (CI 0.89–0.95) for differentiating between the different HCA subtypes using fractal analysis alone.

**Table 3:** Results of global fractal analysis

Lesion entity	*n*	Global FD_diff_	
		Median	Quartiles
H-HCA	13	0.08	0.06–0.10
b^ex3^-HCA	7	0.16	0.15–0.17
I-HCA	27	0.25	0.23–0.31
HCC	16	0.26	0.22–0.30

Global fractal dimension differences (FD_diff_ = FD_tumor_ − FD_liver_) are given as median and quartiles

n, number of patients per lesion entity; HCA, hepatocellular adenoma; H-HCA, HNF1α-inactivated HCA; b^ex3^-HCA, β-catenin-exon-3-mutated HCA; I-HCA, inflammatory HCA; HCC, hepatocellular carcinoma

### Comparison of qualitative features and fractal analysis

Based on qualitative analysis of multiparametric MRI data alone, presence of typical features as outlined above achieved low overall agreement (κ = 0.35 [CI 0.15–0.56]) and moderate correlation (Spearman’s *ρ* = 0.25, *p* = 0.05). Due to missing consensus criteria for typical MRI features in b^ex3^-HCAs, this entity could not be differentiated from visual MRI analysis alone.

Combining visual MRI features and fractal analysis improved the prediction of the histopathological diagnosis to 13/13 H-HCAs (100%), 13/27 I-HCAs (48%), and 16/16 HCCs (100%), and allowed to correctly predict 6/7 b^ex3^-HCAs (86%). Overall, combining visual MRI features with fractal analysis achieved high agreement to histopathological reference (κ = 0.91 [CI 0.83–0.98]) and strong correlation (Spearman’s *ρ* = 0.93, *p* < 0.001).

For differentiating lesion types by clinical relevance, we divided the lesions in our study population into a high-risk group (HCCs and b^ex3^-HCAs) and a low-risk group (H-HCAs and pure I-HCAs) according to their (potential) malignant behavior. As explained in the Methods section, we used an intention-to-diagnose approach to deal with non-diagnostic lesions. Doing so, we found a sensitivity and specificity for identifying high-risk lesions by qualitative MRI features alone of 43% (CI 23–66%) and 47% (CI 32–64%), respectively, with an AUC of 0.55 (CI 0.42–0.68). The combination of qualitative MRI features and fractal analysis achieved 96% (CI 78–100%) sensitivity, 68% (CI 51–81%) specificity, and an AUC of 0.82 (CI 0.73–0.90). This improvement was significant for sensitivity (*p* = 0.003) and AUC (*p* = 0.003) but not for specificity (*p* = 0.3).

## Discussion

Among patients with non-cirrhotic livers, we found that fractal analysis is an effective descriptor of perfusion chaos and adds complementary information for differentiating between HCA subtypes and HCC. When typical qualitative MRI features are absent, fractal analysis can significantly improve diagnostic accuracy in differentiating high-risk (HCC, b^ex3^-HCA) from low-risk lesions (H-HCA, I-HCA). Interestingly, fractal analysis revealed a higher perfusion chaos of HCCs in comparison with b^ex3^-HCAs. Therefore, the FD might be an effective imaging biomarker to differentiate those two entities, for which typical imaging features have not yet been established [6]. In our study, we performed fractal analysis using images acquired with a routine clinical MRI protocol that included multi-phasic dynamic contrast-enhanced imaging during arterial, portal venous, and delayed (3 min) phases. Our reported FD_diff_ values therefore do not require specific protocol adaptations and do not rely on a specific perfusion model.

In clinical practice, correct imaging-based subtyping of HCA is challenging [28] and, although diagnostic criteria exist for most subtypes, individual lesions may present with varying sets of features, which often precludes definitive noninvasive diagnosis [3]. Furthermore, studies using qualitative MRI features for differentiation of HCA subtypes rarely include HCC in non-cirrhotic livers as separate entity. Therefore, the diagnostic performance of MRI features is not well established in such relatively broad populations. Moreover, our study included only lesions with histopathological ground truth, which induced a selection bias, especially for H-HCAs and I-HCAs. The former aspects explain the comparatively low performance of MRI features found in our study and justify exploration of fractal analysis as a quantitative biomarker of perfusion in the challenging population of patients with non-cirrhotic livers.

Fractal analysis allows quantitative assessment of the perfusion pattern, thus providing information on the architecture of the underlying vasculature. Differences in vascular structure have been found between different HCA subtypes [31]. In our study, FD_diff_ was similar in I-HCA and HCC, which both had highly chaotic perfusion patterns on visual inspection. This observation might be attributable to high vascular density and potentially similar architectural vascular changes due to inflammation or tumor angiogenesis, especially in comparison with benign lesions like b^ex3^-HCA prior to malignant transformation. Indeed, compared to the other subtypes of adenomas like b^ex3^-HCA, I-HCA contains more arteries and dilated sinusoids which are commonly of large size, and HCC is composed of numerous isolated arteries [32, 33]. Such differences in vascular architecture stimulated the present study, and our results suggest that characterization of perfusion patterns is suitable to quantitatively assess the relationship between contrast agent deposition and the underlying vascular structure.

Since perfusion is inherently chaotic, an absolute interpretation of the FD can be challenging, especially in the liver, with its unique dual vascular supply by the arterial and portal venous system. In previous studies, quantitative intensity measurement has been standardized to normal liver parenchyma [28]. We adopted this approach and individually calibrated quantitative FD values according to the physiological level of perfusion chaos in each patient, and we employed individual noise level and intensity-adapted preprocessing to standardize all imaging sequences. Differences in global FD determined in this way were thus found to reliably differentiate the tumor entities investigated in this study. Since the patients included in our analysis were examined on two different MRI scanners from different manufactures with administration of different contrast agents, our approach allowed us to standardize quantitative measurement, thus creating a biomarker that yields consistent and reliable results in different imaging setups. Even if hepatospecific GD-BOPTA contrast agent differs from the extracellular GD-DOTA contrast agent in terms of pharmacokinetics, the hepatobiliary phase of GD-BOPTA occurs late (at least 60 min after administration). Therefore, the three-minute delayed phase can be considered to be similar to that obtained with an extracellular contrast agent [34].

This study has limitations: The number of cases included in this retrospective analysis is limited, and no shHCA or b-HCA with confirmed exon7,8-mutation were included. These are two rare subtypes, each accounting for approximately 4% of all HCAs [3]. Moreover, hybrid or unclassified HCA subtypes were not present in the study population. Those subtypes are not yet well understood and may require further histopathological and genetical insights before fractal analysis becomes meaningful. As discussed above, the inclusion of different HCA subtypes and HCC in normal livers without risk factors for HCC development led to a selection bias linked to biopsy or resection, as lesions without histological ground truth (albeit potentially typical imaging appearance) were excluded. Due to biopsy availability, only one lesion per patient with histological subtyping was included in the analysis; therefore, we cannot conclude on subtypes of not biopsied and, hence, not analyzed lesions in patients with multiple lesions. The imaging protocol might have confounded fractal analysis results, specifically because we decided to include MRI examinations performed on both 1.5 T and 3 T scanners and because experimental validation in an immediate scan–rescan experiment using different field strengths is not yet available for fractal analysis. However, given the scale-invariant nature and since we implemented a preprocessing scheme to account for noise and image signal normalization, we expect effects of field strength on fractal analysis to be minimal. Moreover, we standardized fractal analysis results using remote liver tissue sampled from adjacent locations to minimize the confounding effect of local field strength inhomogeneities. Furthermore, no conclusion can be drawn on the diagnostic value of fractal analysis in the cirrhotic liver because prospective data to independently validate our established thresholds are not yet available.

Our results might stimulate further research to monitor potential malignant transformation of lesions in patients managed by watchful waiting. Fractal analysis might constitute an indicator for such malignant transformation and might be useful in identifying lesions that require definitive surgical treatment versus lesions that only require follow-up. Moreover, it might be interesting to investigate whether our results could also help in predicting major complications, e.g., hemorrhage or rupture, which are more common and potentially life-threatening complications in HCAs [35]. As a quantitative imaging method, diffusion-weighted imaging (DWI) has shown a large overlap of visual appearance and quantitative values in focal liver lesions [36]; however, it might be interesting to investigate its value when combined with fractal analysis of perfusion. Finally, prospective investigation of the clinical benefit of fractal analysis with suspension of biopsy for HCA diagnosis in a controlled study setting might be valuable.

## Conclusions

In conclusion, our study indicates that the chaos of perfusion differs between HCA subtypes and between HCA and HCC in the non-cirrhotic liver. Fractal analysis can be used to quantify these differences using three-phasic 4D DCE-MRI. According to our hypothesis, chaos of the perfusion pattern is strongly related to the organization of the underlying vascular structure, which is assumed to differ between the tumor entities and subtypes analyzed in our study.

## Supplementary Information

**Additional file 1.** Image Preprocessing.

## Data Availability

The datasets used and/or analyzed during the current study are available from the corresponding author on reasonable request.

## Abbreviations

APHE: Arterial phase hyperenhancement
AUC: Area under the receiver operating characteristic curve
b^ex3^-HCA: β-Catenin-exon-3-mutated hepatocellular adenoma
CI: 95%-Confidence interval
DCE MRI: Dynamic contrast-enhanced magnetic resonance imaging
EASL: European Association for the Study of the Liver
FD: Fractal dimension
HCA: Hepatocellular adenoma
HCC: Hepatocellular carcinoma
H-HCA: Hepatocyte nuclear factor-1α-inactivated hepatocellular adenoma
HNF: Hepatocyte nuclear factor
I-HCA: Inflammatory hepatocellular adenoma
shHCA: Sonic hedgehog hepatocellular adenoma
