# Supplementary material for: Differentiation of hepatocellular adenoma by subtype and hepatocellular carcinoma in non-cirrhotic liver by fractal analysis of perfusion MRI
Source: Insights Imaging. 2022 Apr 28;13:81. doi: 10.1186/s13244-022-01223-6 (PMC9050986; doi:10.1186/s13244-022-01223-6)
Supplement: Supplementary file 1 — Additional file 1. Image Preprocessing. [file 13244_2022_1223_MOESM1_ESM.docx]

**ELECTRONIC SUPPLEMENTAL MATERIAL**

*Image Preprocessing*

Image preprocessing was performed prior to fractal analysis and consisted of the following steps: (i) multi-phase registration, (ii) image denoising, (iii) intensity standardization, and (iv) segmentation. (i) The 4D DCE sequences were registered using the SimpleElastix framework (Marstal K. 2015. SimpleElastix. <https://github.com/SuperElastix/SimpleElastix>). We employed a 4D multi-resolution registration approach based on principal component analysis (PCAmetric2) with elastic b-spline-based transformation. (ii) Image data were denoised using a median filter with a 1x1x1 mm kernel and a bilateral filter with a 1x1x1 mm spatial kernel and an individually determined value for the intensity domain to accomplish noise-level-adapted denoising. The latter was obtained from measurement of the standard deviation in the erector spinae muscle on the unenhanced phase. This denoising scheme ensured consistent image quality by considering the individual noise level of each imaging dataset. (iii) Image intensity was standardized relative to the signal in the portal vein by the following formula: I_stnd_(x,y,z,t) = I(x,y,z,t)_scanner_ / I_max,pv_ * 100, where I_stnd_ and I_scanner_ denote signal intensity after and before standardization, respectively, and I_max,pv_ denotes the maximum signal in the portal vein for that time series. (iv) Segmentation was performed in a standardized semiautomatic manner for both the liver lesion and remote normal liver parenchyma. Due to their ovoid shape, lesion segmentation was straightforward using sparse 2D segmentation with volume interpolation and a final visual check. Consistent segmentation results were achieved, and no manual correction was necessary. Remote normal liver tissue was selected using an ovoid shape placed in a location adjacent to the respective lesion in the same liver lobe with careful avoidance of partial volume from extra-hepatic tissue and large liver vessels.

**Supplemental Table 1.** Immunohistochemical and molecular diagnosis of each hepatocellular adenoma (HCA).

| **lesion**  **number** | **Resected**  **1=yes**  **0=no** | **Number of biopsy samples** | **Diagnosis** | **SAA** | **CRP** | **GS pattern** | **B-catenin pattern** | **LFABP pattern** | **PTGDS** | **Molecular analysis*** |
| --- | --- | --- | --- | --- | --- | --- | --- | --- | --- | --- |
| 1 | 1 |  | Ex3-B-cat | - | - | Diffuse homogeneous | Nuclear |  | - |  |
| 2 | 1 |  | Ex3-B-cat | - | + | Diffuse homogeneous | Nuclear | maintained | - | CTNNB1 ex-3 mutations |
| 3 | 0 | 4 | Ex3-B-cat | - | - | Diffuse homogeneous | Nuclear |  |  |  |
| 4 | 1 |  | Ex3-B-cat | - | - | Diffuse homogeneous | Nuclear |  |  |  |
| 5 | 1 |  | Ex3-B-cat | - | - | Diffuse homogeneous | Nuclear | maintained | - | CTNNB1 ex-3 mutations |
| 6 | 1 |  | Ex3-B-cat | - | - | Heterogenic | Nuclear |  |  | CTNNB1 ex-3 mutations |
| 7 | 0 | 3 | Ex3-B-cat | - | - | Diffuse homogeneous | Nuclear | maintained |  |  |
| 8 | 1 |  | IHCA | + | + | Perivascular | Membrane | maintained |  |  |
| 9 | 1 |  | IHCA | + | + | Negative |  | maintained |  |  |
| 10 | 0 | 2 | IHCA | + | + | Negative |  | maintained |  |  |
| 11 | 0 | 3 | IHCA | - | + | Negative |  | maintained |  |  |
| 12 | 1 |  | IHCA | + | + | Perivascular | Membrane | maintained |  |  |
| 13 | 0 | 1 | IHCA | + | + | Perivascular | Membrane |  |  |  |
| 14 | 0 | 2 | IHCA | + | + | Negative |  |  |  |  |
| 15 | 0 | 2 | IHCA | - | + | Negative | Membrane | maintained |  |  |
| 16 | 0 | 3 | IHCA | + | + |  | Membrane |  |  |  |
| 17 | 0 | 2 | IHCA | + | + | Perivascular | Membrane |  |  |  |
| 18 | 1 |  | IHCA | - | + | Heterogenic | Membrane |  |  | JAK1 mutation |
| 19 | 0 | 1 | IHCA | - | + | Negative | Membrane |  |  |  |
| 20 | 1 |  | IHCA | + | + | Perivascular |  |  |  |  |
| 21 | 0 | 3 | IHCA | + | + | Perivascular |  |  |  |  |
| 22 | 0 | 2 | IHCA | - | + | Negative |  |  |  |  |
| 23 | 0 | 1 | IHCA | - | + | Negative |  | maintained |  |  |
| 24 | 0 | 4 | IHCA | + | + | Negative | Membrane |  |  |  |
| 25 | 0 | 3 | IHCA | + | - | Perivascular | Membrane |  |  |  |
| 26 | 1 | 4 | IHCA | + | + | Perivascular |  |  |  |  |
| 27 | 0 | 2 | IHCA | + | + | Focal | Membrane |  |  |  |
| 28 | 1 |  | IHCA | + | + | Perivascular |  |  |  |  |
| 29 | 0 | 2 | IHCA | + | + | Perivascular | Membrane |  |  |  |
| 30 | 0 | 4 | IHCA | + | - | Perivascular | Membrane |  |  |  |
| 31 | 0 | 4 | IHCA | + | + | Negative |  |  |  |  |
| 32 | 0 | 3 | IHCA | + | + | Perivascular |  |  |  |  |
| 33 | 0 | 3 | IHCA | - | + | Perivascular | Membrane | maintained | - |  |
| 34 | 0 | 2 | IHCA | + | + | Perivascular |  |  |  |  |
| 35 | 0 | 2 | HHCA | - | - |  |  | lost |  |  |
| 36 | 0 | 2 | HHCA | - | - |  |  | lost |  |  |
| 37 | 1 |  | HHCA | - |  |  |  | lost | - | HNF1A mutation |
| 38 | 0 | 4 | HHCA |  |  |  | Membrane | lost | - |  |
| 39 | 1 |  | HHCA | + | - |  | Membrane | lost |  |  |
| 40 | 0 | 5 | HHCA | - | - | Negative |  | lost | - |  |
| 41 | 0 | 2 | HHCA |  |  |  |  | lost |  |  |
| 42 | 0 | 3 | HHCA |  |  |  |  | lost |  |  |
| 43 | 0 | 4 | HHCA | - | - |  |  | lost |  |  |
| 44 | 0 | 2 | HHCA | - | - |  |  | lost |  |  |
| 45 | 0 | 2 | HHCA |  |  |  |  | lost |  |  |
| 46 | 0 | 3 | HHCA | - | - | Perivascular |  | lost |  |  |
| 47 | 0 | 3 | HHCA | - | - |  |  | lost |  |  |

Immunohistochemical markers were performed according to the morphological aspect of the lesion. Molecular analysis was only performed in cases difficult to classify with morphological and immunohistochemical analyses. CRP: C-reactive protein; Ex3-BHCA: exon-3 β-catenin mutated HCA; GS: glutamine synthetase; IHCA: Inflammatory HCA; HHCA: HNF1A-inactivated HCA; LFABP: liver fatty acid binding protein; PTGDS: prostaglandin D synthase; SAA: serum amyloid A; *: pathognomonic mutation is reported; +: positive; -: negative; empty cell: not performed.

**Supplemental Table 2.** Imaging protocol details.

| **Name of MRI device** | **Philips Achieva 3T** | **GE SIGNA 1.5T** |
| --- | --- | --- |
| Pulse sequence | Axial T1-weighted fat-suppressed spoiled gradient-recalled echo sequence | Axial T1-weighted fat-suppressed spoiled gradient-recalled echo sequence |
| Number of slices | 90 | 90 |
| TR/TE (ms) | 4.6/2.2 | 3.8/1.8 |
| Flip angle (°) | 10 | 12 |
| Slice thickness (mm) | 3 | 3.5 |
| Matrix size | 240x240 | 256x256 |
| Number of excitations | 2 | 0.7 |

**Supplemental Figure 1.** Bland-Altman plot for inter-reader agreement of fractal analysis.


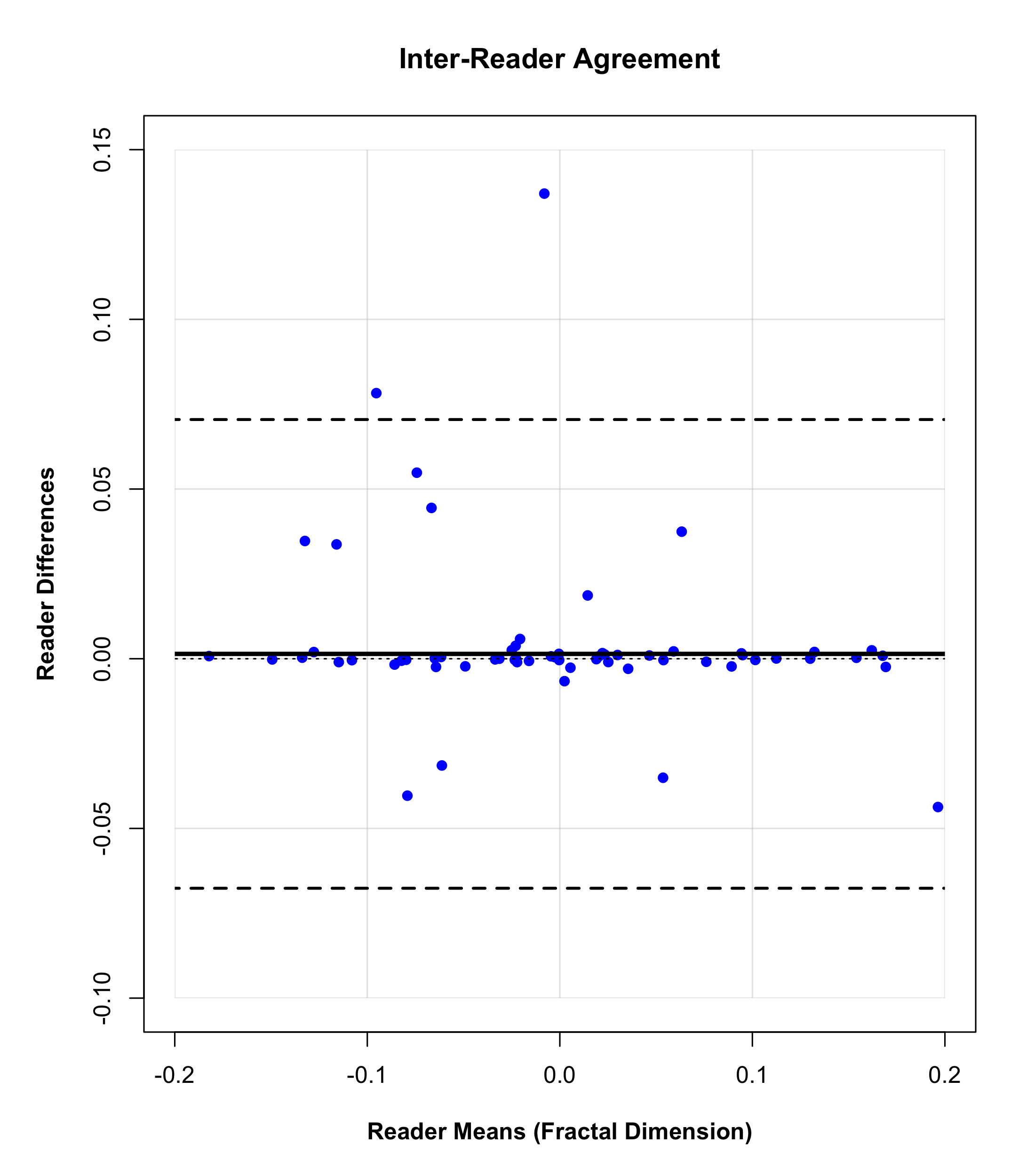


Inter-reader agreement of quantitative fractal dimension results was high, which can be explained by the automated and standardized image preprocessing pipeline and automated calculation of fractal dimension as explained in the Methods section. The remaining variability might be explained by segmentation variations.
